# Intradiscal Injections of Bone Marrow Concentrate or Leukocyte-Rich Platelet-Rich Plasma for the Treatment of Cervical Discogenic Pain: A Case Series

**DOI:** 10.7759/cureus.84166

**Published:** 2025-05-15

**Authors:** John Pitts, Jason Markle, Dustin Berger, Ehren Dodson

**Affiliations:** 1 Interventional Pain Management, Centeno-Schultz Clinic, Broomfield, USA; 2 Physical Medicine and Rehabilitation, Interventional Orthopedics and Regenerative Medicine, Centeno-Schultz Clinic, Broomfield, USA; 3 Research and Development, Regenexx, Broomfield, USA

**Keywords:** bone marrow concentrate (bmc), cervical intradiscal, functional spinal unit (fsu), interventional pain management, neck pain, orthobiologics, platelet lysate (pl), platelet-rich plasma (prp), regenerative medicine therapies

## Abstract

Purpose: Cervical discogenic pain is a prevalent and debilitating condition. In recent years, autologous regenerative therapies such as bone marrow concentrate (BMC) and platelet-rich plasma (PRP) have gained attention as potential alternatives to traditional interventional pain management. This study investigates the safety and efficacy of these treatments when delivered intradiscally as part of a comprehensive cervical functional spinal unit (FSU) approach.

Methods: This is a retrospective case series utilizing registry data, with 18 participants meeting the inclusion criteria. Intradiscal injections of BMC or leukocyte-rich PRP were guided by ultrasound and fluoroscopy with contrast confirmation. Patient-reported outcome measures (PROMs), including the Functional Rating Index (FRI), Numeric Pain Scale (NPS), and a modified Single Assessment Numeric Evaluation (SANE), were collected at baseline and at a minimum of three months post-treatment, with a mean follow-up of one year.

Results: Eleven participants received BMC injections, and seven received leukocyte-rich PRP injections. No adverse events were reported during the study period. Statistical analysis revealed a significant reduction in the NPS in half of the patients (9 of 18) (mean difference = −1.0, P = 0.024), while a majority (14 of 18) reported improvement in FRI scores (mean difference = −10.9, P = 0.015). The average SANE score was 60%, with most patients (14 of 18) reporting 50% or greater improvement.

Conclusion: Intradiscal injections of BMC or LR-PRP as part of a comprehensive cervical FSU approach appear to be safe and effective for improving patient-reported pain and function. These findings support the potential of autologous biologics as an alternative treatment modality for cervical discogenic pain.

## Introduction

Neck pain is a significant musculoskeletal problem, with a one-year incidence between 10.4% and 21.3% [1]. Its worldwide prevalence is increasing, especially in high-income countries such as those in North America, Western Europe, and East Asia [2]. One of the contributors to pain in the cervical region is discogenic pain, with a reported prevalence of 16% to 41% in those with neck pain [3]. Anatomical studies have shown that the C5-C6 and C6-C7 discs are most commonly affected. Additionally, as described by Cloward et al., cervical discogenic pain may have atypical referral patterns observed below the pathological disc level and into the periscapular region [4,5]. The etiology of discogenic pain syndrome can include trauma, rapid unexpected movements, forward head posture, and chronic neck flexion [6]. Symptoms and exam findings can vary but often include pain with sitting, flexion, head protrusion, heavy lifting, or upon awakening in the morning from awkward sleeping positions [6]. Imaging may show cervical disc annular tears, protrusions, and/or degeneration, but there is no strong correlation between magnetic resonance imaging (MRI) findings and symptoms [3]. Cervical discography can assist in diagnosing discogenic pain, although it has variable accuracy and inherent risks as a strictly diagnostic procedure [6].

Treatment of cervical discogenic pain typically begins with conservative management, including physical therapy and either over-the-counter or prescription-strength medications (e.g., muscle relaxants or opioids). If conservative measures fail, interventional pain management is often recommended, such as corticosteroid injections or radiofrequency ablation, prior to considering surgical intervention [6]. Recently, orthobiologic injections using autologous preparations, including platelet-rich plasma (PRP) and bone marrow concentrate (BMC), have been used to treat musculoskeletal conditions. Previously published studies have demonstrated long-term improvements in pain and functional outcomes in patients treated with lumbar intradiscal PRP and BMC for discogenic pain after failure of conservative measures [7,8]. To our knowledge, only two single-patient case studies have described different approaches for injecting PRP intradiscally under image guidance for cervical discogenic pain [9,10], and a third study described PRP injection into the cervical facets following whiplash injury [11]. Previously, our group reported on treatment of the cervical spine with PRP, platelet lysate (PL), and prolotherapy using a functional spinal unit (FSU) approach, which showed improved pain and functional outcomes [12]. The FSU approach involves the consideration and treatment of multiple tissue types, including the facet joints, ligaments, nerves, and tendons/muscles [12]. In this case series, we present registry data from patients treated using the FSU approach with the addition of an intradiscal injection of BMC or highly concentrated leukocyte-rich PRP.

## Materials and methods

Patient selection

Patients were treated at a single-center, outpatient-based interventional pain center by authors JP or JM. All consenting patients were prospectively enrolled into the patient registry following an Institutional Review Board-approved registry protocol (HHS OHRP #IRB00002637). The electronic registry collects baseline demographics, clinical outcomes, and adverse events with follow-up surveys at 1, 3, 6, 12, 18, and 24 months, and annually thereafter, for up to 20 years (Dacima Software, Montreal, Quebec; and later Salesforce Software, San Francisco, California). Up to five attempts are made to collect data at each follow-up time point. Inclusion criteria for this analysis included patients with axial neck and periscapular pain, with or without radiculopathy, and suspicion of cervical discogenic pain that had failed at least three months of prior conservative care (i.e. physical therapy, medical management such as acetaminophen, NSAIDs (nonsteroidal anti-inflammatory drugs), gabapentin, and in some cases, corticosteroid injections). All cervical MRIs showed an annular tear, disc protrusion, and/or disc bulge. Patients were treated via image-guided injections of leukocyte-rich PRP or BMC into cervical disc(s), in addition to injections of PRP or PL to cervical facet joints, and/or PL to cervical transforaminal epidural injections, and/or PRP or prolotherapy to cervical ligaments between March 2018 and January 2023. Post-procedure, patients were given a few days’ worth of narcotics for post-procedural pain management. All patients were advised to restart or continue physical therapy post-procedure, but this was not controlled. Only patients with available pre- and post-treatment registry outcome data after a minimum of three months were included. Search criteria to identify patients within the registry included those who underwent cervical intradiscal procedures, before a more extensive chart review was conducted to confirm inclusion criteria. Patients were excluded if they had a concurrent diagnosis of cranial cervical instability requiring interventional treatment.

Patient-Reported Outcome Measures (PROMs)

Cervical spine clinical outcomes were measured by several patient-reported questionnaires, including the Numeric Pain Score (NPS), Functional Rating Index (FRI), and a modified Single Assessment Numeric Evaluation (SANE) [13-15]. The NPS is a scale ranging from 0 to 10, where 0 equates to no pain and 10 equates to the worst possible pain. The FRI measures perceived dysfunction and pain on a scale of 0 to 100%, where 0 reflects no disability and 100% represents very severe disability. The modified SANE asked patients to rate, on a scale from -100 (worsened) to +100 (improved), the percentage difference they felt post-treatment compared to pre-treatment. Minimal clinically important difference (MCID) values of -2, -9, and 10.5% were used for the NPS, FRI, and SANE scores, respectively [16-18].

Preparation of PRP and PL

In preparation for treatment, patients were instructed to cease the use of NSAIDs and aspirin one week prior to minimise interference with platelet activity, and oral or injected corticosteroids six weeks prior to mitigate any catabolic and anti-inflammatory effects. Within 24 hours of the treatment procedure, patients underwent a venous blood draw ranging from 50 mL to 500 mL, depending on the number of structures being injected. Blood was drawn into glass tubes (8.5 mL per tube) or blood bags (500 mL per bag) with anticoagulant citrate dextrose solution A (ACD-A) mixed in (1.5 mL per tube or 80 mL per bag). All blood products were manually processed utilising laboratory aseptic technique within laminar flow cabinets (ISO-5) in a sterile clean room (ISO-7), the details of which have been previously described [19,20].

In brief, to prepare leukocyte-rich PRP (LR-PRP) for intradiscal applications, whole blood was centrifuged at 200×g to separate the plasma and buffy coat fractions from the underlying red blood cells. Isolated plasma and buffy coat layers were recentrifuged to pellet the cell and platelet bodies, which were resuspended in a final plasma volume one-tenth that of the initial plasma volume. Leukocyte-poor PRP (LP-PRP), for treating the FSU, was prepared using a similar method, with care taken to avoid capturing the buffy coat. A portion of PRP was frozen at -80°C and then thawed to prepare PL. The final product of prepared PRP and PL was placed into a sterile bag until utilisation for the procedure.

Bone Marrow Aspiration and Preparation of BMC

Whole bone marrow aspirate (BMA) was harvested via an 11-gauge trocar from both sides of the patients' posterior superior iliac crest under ultrasound guidance, utilizing surgical sterile technique. Approximately 10 to 15 mL of BMA was withdrawn from each of six to eight sites (three to four per side) into heparinized syringes containing 1,000 units of heparin per mL of BMA obtained. Thereafter, BMA was processed utilizing laboratory aseptic technique within laminar flow cabinets (ISO-5) in a sterile cleanroom (ISO-7) to isolate BMC. Briefly, BMA (60 to 120 mL) was centrifuged at 200×g for six minutes to form three distinct fractions: a lower fraction largely containing red blood cells, an upper fraction of acellular plasma, and at the interface, a middle fraction rich in nucleated cells, also known as the buffy coat. This middle fraction was manually isolated via pipette and then recentrifuged at 200×g for six minutes. Again, the final concentrated buffy layer was isolated via pipette, producing one to eight mL of BMC injectate. A small representative sample (<0.1 mL) was reserved for obtaining a cell count with an automated hematological analyzer (Micros60; Horiba, Montpellier, France), using the white blood cell parameter as a measure for total nucleated cells. The final product was transported via sterile means back to the procedural room prior to injection.

Cervical Intradiscal Injection Technique

Under surgically sterile conditions, all patients received percutaneous injection of the desired disc(s) between the levels of C2-3, C3-4, C4-5, C5-6, and C6-7, using ultrasound and multiplanar fluoroscopic guidance. All injections were performed and developed by authors JP and JM. Two procedural techniques were developed: a preferred approach and an alternative approach, utilized if a particular patient’s anatomy made it difficult or unsafe to perform the former. Patients were sedated via total intravenous anesthesia with midazolam and fentanyl for comfort and safety.

**Figure 1:**
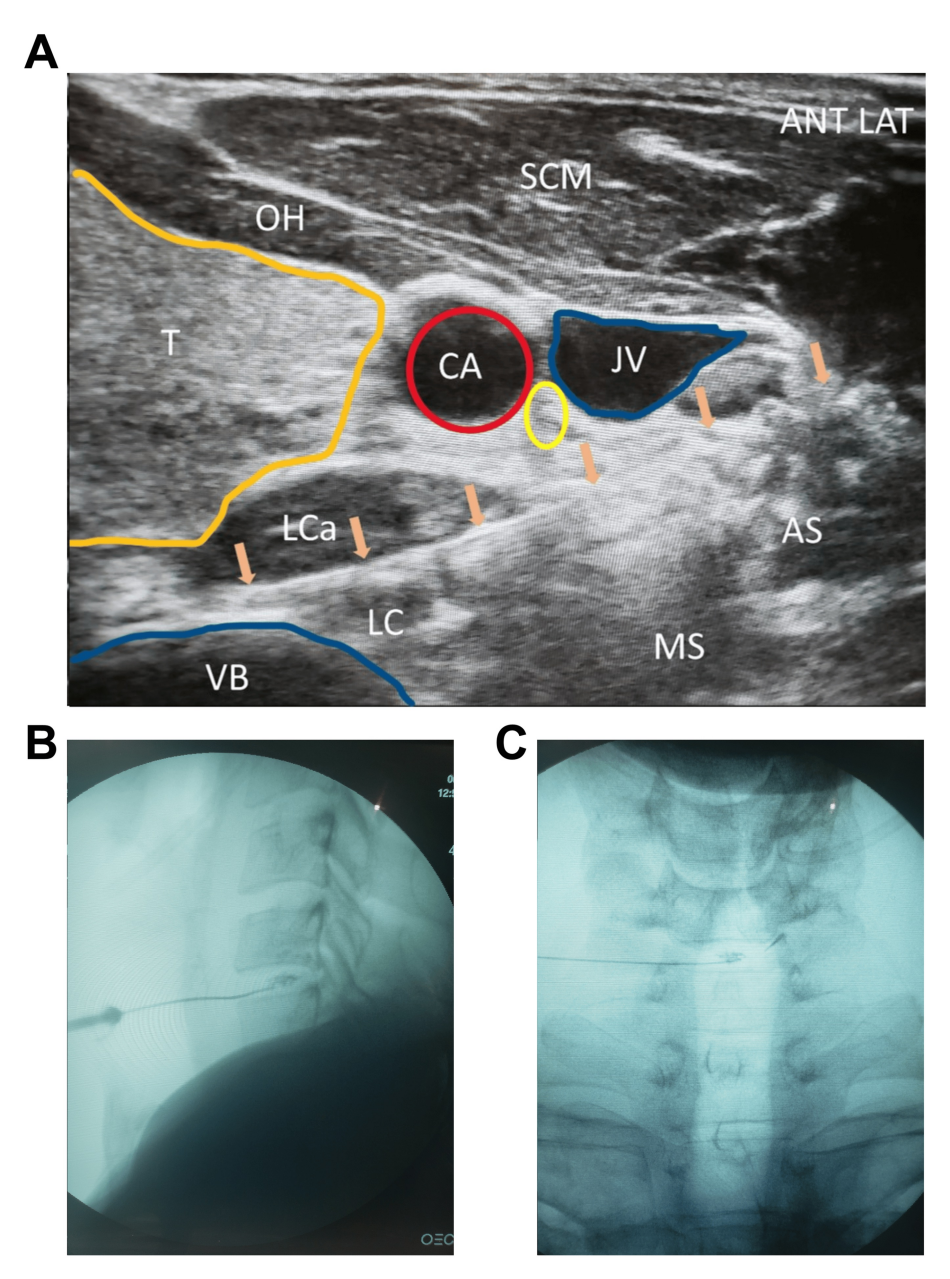
Cervical Intradiscal Injection Technique. Needle advanced deep to the great vessels under linear in-plane ultrasound guidance. Arrows indicate needles. Yellow circle denotes vagus nerve. ANT LAT, anterior lateral; T, thyroid; CA, carotid artery; JV, jugular vein; VB, cervical vertebral body; LC, longus colli; LCa, longus capitis; AS, anterior scalene; MS, middle scalene; OH, omohyoid.

Preferred approach: The preferred approach involved positioning the patient supine, with the head in slight extension and approximately 30 degrees rotation to the left. The proceduralist stood on the right side of the patient, and the needle was always inserted from the right to avoid the esophagus on the left. A linear ultrasound probe was used to scan the neck and identify the carotid artery, jugular vein, vertebral artery, nerve roots, vertebral bodies, and intradiscal space. A 25-gauge spinal needle was used with a slight bend of the tip away from the bevel to assist with angling the needle posteriorly into the disc. The needle was guided under ultrasound below or through the internal jugular vein, depending on the angle needed to access the disc while avoiding the uncovertebral joint (Figure 1A). Care was taken to avoid the carotid artery, vertebral artery, and nerve roots. The needle passed through the sternocleidomastoid (SCM), anterior scalene, and longus coli muscles. Once the needle tip was past the great vessels and close to the disc, a true AP fluoroscopic image was taken to confirm the level of the target disc space and assess whether uncinate processes or bone spurs needed to be avoided. The needle was then guided into the center of the disc nucleus. Anteroposterior (AP) and lateral fluoroscopic views were used to confirm accurate needle placement. If the needle was not in the nucleus, adjustments were made if possible; otherwise, a new angle or the alternative approach was used. Visipaque contrast (0.1 mL) was injected to confirm a nucleogram and to visualize flow into the annulus or evidence of extravasation (Figure 1B-C). After contrast confirmation, 0.5 mL of PRP or BMC was injected into the nucleus and into the punctured annulus. The needle was then removed.

**Figure 2:**
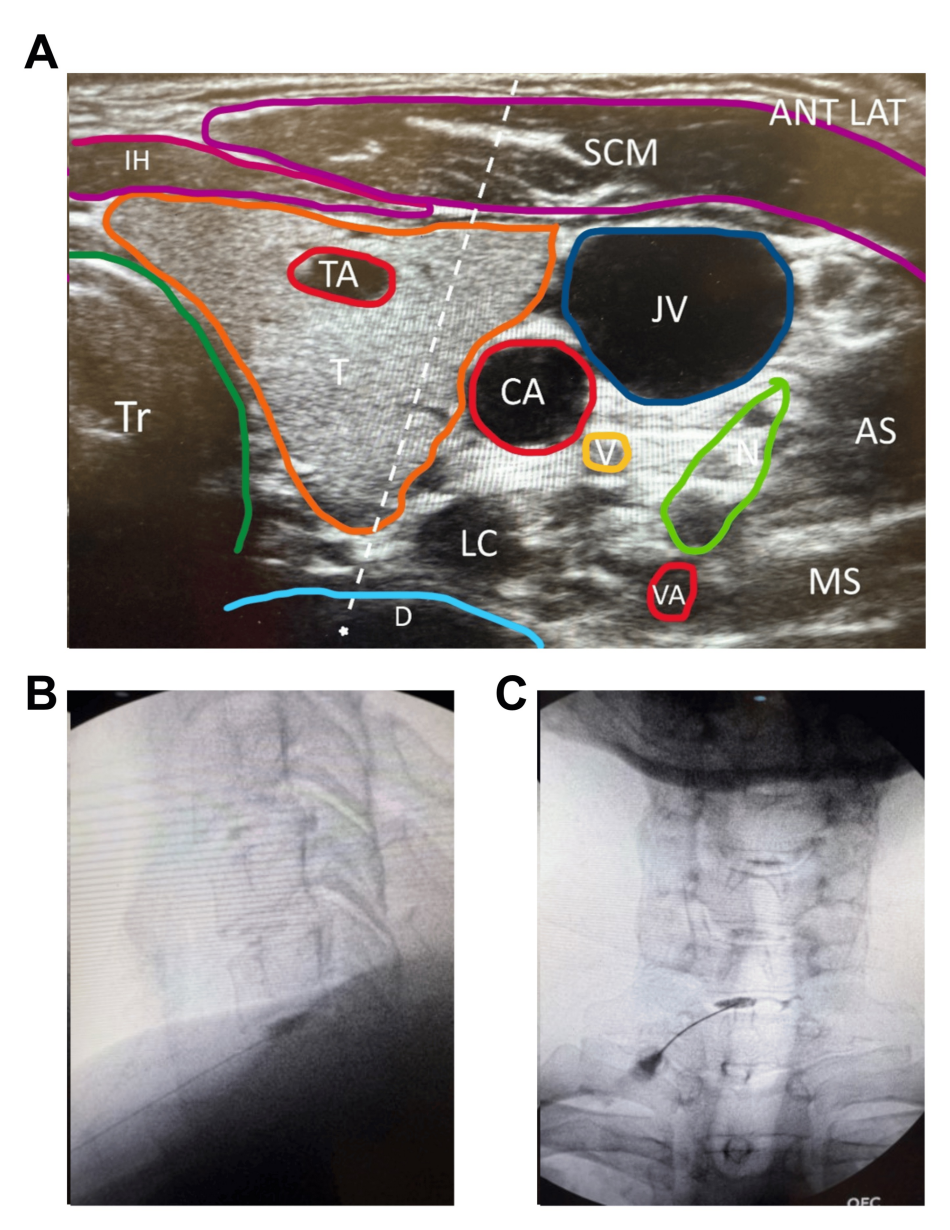
Cervical Intradiscal Alternative Injection Technique. Needle advanced between the thyroid artery and carotid artery using an out-of-plane approach. Green circle: nerve roots; dashed lines: needle trajectory; star: needle target. ANT LAT, anterior lateral; T, thyroid; Tr, trachea; CA, carotid artery; JV, jugular vein; SCM, sternocleidomastoid; IH, infrahyoid muscles; TA, thyroid artery; LC, longus colli; AS, anterior scalene; MS, middle scalene; D, intervertebral disc; V, vagus nerve.

Alternative approach: The same patient positioning, linear probe, and setup as described above were used. In this technique, an out-of-plane injection approach was employed, guiding the needle medial to the carotid artery and lateral to the thyroid artery (Figure 2A). The ultrasound probe was placed transversely across the neck to identify the desired disc level. An AP fluoroscopic view could be used to confirm the disc level. Color Doppler was utilized to identify any additional blood vessels that may be present along the needle trajectory, as the 25-gauge, 3-inch needle was guided out of plane through the thyroid while avoiding the carotid and thyroid arteries. Fluoroscopy was subsequently used to advance the needle into the disc nucleus, with confirmation of placement through AP and lateral views and by evaluating the contrast flow pattern (Figure 2B-C). The contrast and injectate volumes were the same as described above.

Cervical FSU Injections Technique

**Figure 3:**
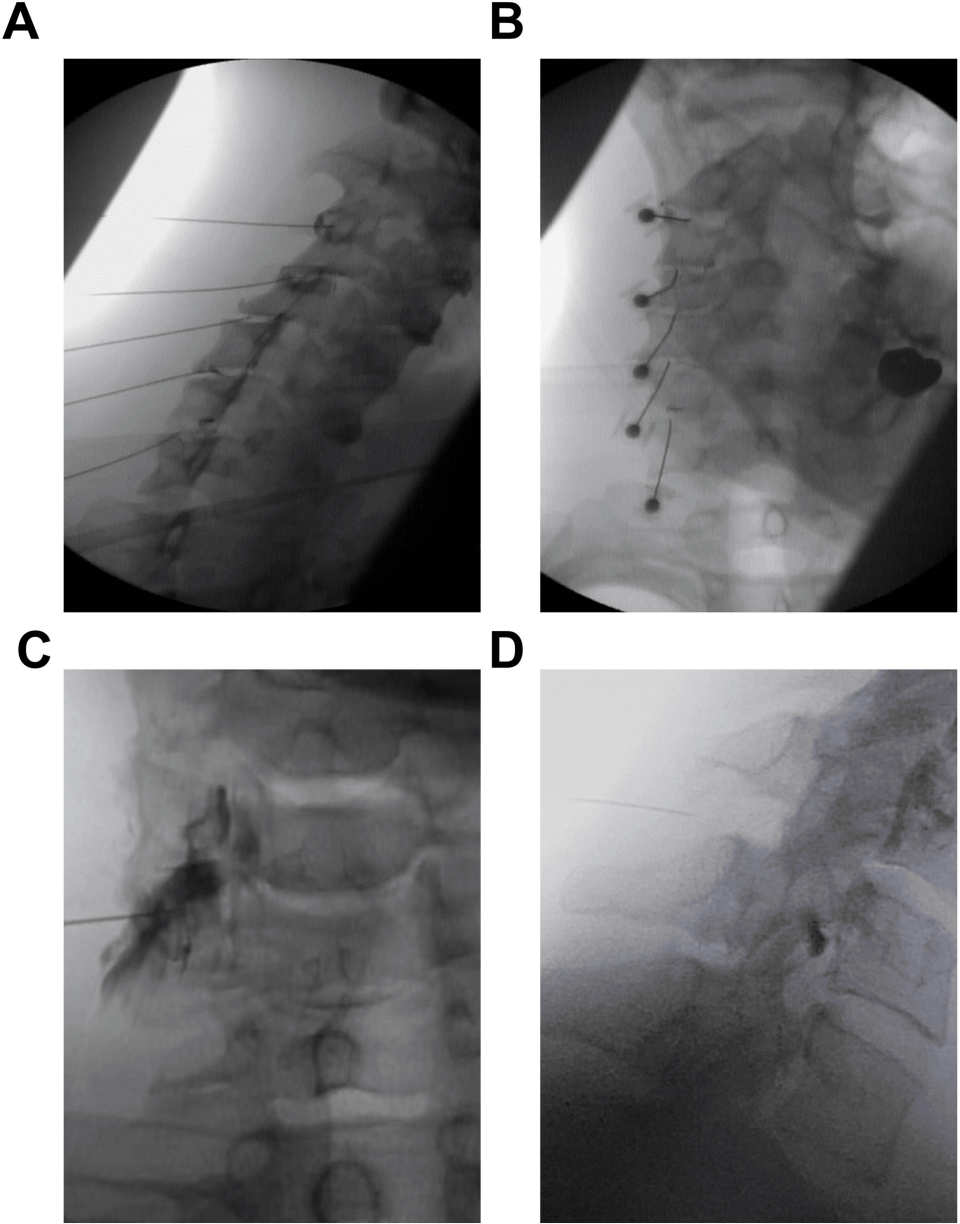
Cervical Functional Spinal Unit (FSU) Injection Technique. Contralateral oblique view of intra-articular facet injection with contrast confirmation (A); anteroposterior view of intra-articular facet injection with contrast (B); anteroposterior view of transforaminal epidural injection with contrast confirmation (C); and lateral view showing needle placement for interspinous ligament injection (D).

All patients received a combination of cervical facet joint injections, cervical ligament injections, and/or transforaminal epidural injections (Figure 3A-D). Specific injection levels and laterality were determined based on symptoms, physical examination, and imaging studies. Cervical facet injections were performed under multiplanar fluoroscopic guidance using the posterior approach. Intraarticular flow was confirmed with contrast. Following this, cervical supraspinous and interspinous ligament injections were performed under fluoroscopic guidance, utilizing the lateral view for needle placement and using the spinous process to safely determine needle depth. At each level of interest, the needle was directed both above and below the spinous process, with advancement posterior to the spinolaminar line. Cervical transforaminal injections were performed in the supine position via an anterolateral approach under multiplanar fluoroscopic guidance. A 25-gauge spinal needle was advanced under ipsilateral oblique view to the posterior foramen. In the AP view, the needle was positioned at the lateral third of the lateral mass. Contrast was injected to confirm epidural spread and absence of vascular uptake. Facet joints were injected with 0.5 to 1 mL of leukocyte-poor PRP, PL, or BMC. Cervical transforaminal epidurals were injected with 1 mL of ropivacaine and 2 mL of PL. Cervical supraspinous and interspinous ligaments were injected with a total of 5 to 10 mL of a BMC and PRP mixture, PRP alone, prolotherapy (12.5% dextrose, ropivacaine, saline), or prolotherapy combined with PL (in place of saline).

Statistical Analysis

Patient demographic and PROM data were confirmed to be normally distributed using the D’Agostino and Pearson test, and descriptive statistics for continuous variables were presented as mean and standard deviation. Individual differences between the most recent post-treatment follow-up time point and baseline PROMs (termed “change scores” or ΔPROMs) were calculated for NPS and FRI metrics. Paired t-tests were used to assess whether post-treatment scores differed significantly from baseline. P-values below 0.05 were considered statistically significant. All analyses were performed using GraphPad Prism 10.2 (GraphPad Software, Boston, Massachusetts).

## Results

A total of 52 patients were identified in the registry as having received cervical intradiscal injections. Of those patients, 18 (5 female and 13 male) had baseline measures and at least one follow-up with completed PROMs three months or longer post-treatment. Patient ages ranged from 33 to 73 years (54.8 ± 11.4 years). Additional patient demographics are presented in Table 1. A summary of cervical discs treated, injectates used, BMC cell counts, and PROMs, including NPS, FRI, and SANE scores, is presented in Table 2. A single-level intradiscal injection was performed in eight patients, two-level injections in eight patients, and three-level injections in two patients. The most commonly injected cervical disc was C5-6 (13 of 18 patients), followed by C4-5 (7 patients) and C6-7 (7 patients).

**Table 1:** Summary of Patient Demographic Information. Values represent mean ± standard deviation.

Variables	Values
Gender (n)	18 (5 female and 13 male)
Age (years)	54.8 ± 11.4
Height (inches)	69.1 ± 3.7
Weight (lb)	168.1 ± 25.6
BMI (kg/m^2^)	24.5 ± 2.2

**Table 2:** Summary of Cervical Discs Treated, Injectates, BMC Cells, PROMs and Durations Between Baseline and Last Follow-up. LR-PRP: leukocyte-rich platelet-rich plasma, BMC: bone marrow concentrate, NPS: numeric pain score, FRI: functional rating index, SANE: single assessment numeric evaluation, PROMs: patient-reported outcome measures.

Patient	Treated Cervical Disc Levels	Intradiscal Injectate	Cells (10^6^/disc)	Pre NPS	Post NPS	Pre FRI	Post FRI	Post SANE	Pre to Post (months)
A	Two (C5-6, C6-7)	LR-PRP	-	7	4	72.5	62.5	75	3
B	Two (C4-5, C5-6)	LR-PRP	-	1	0	12.5	0	90	24
C	One (C4-5)	BMC	190	3	0	30	0	100	12
D	Two (C4-5, C5-6)	BMC	82	7	2	60	25	15	20
E	One (C6-7)	BMC	54	1	1	-	-	90	24
F	Two (C5-6, C6-7)	LR-PRP	-	1	1	17.5	15	85	3
G	One (C5-6)	LR-PRP	-	2	0	32.5	5	90	18
H	Two (C5-6, C6-7)	LR-PRP	-	6	4	47.5	35	50	6
I	One (C5-6)	BMC	88	3	0	37.5	25	75	12
J	One (C7-T1)	BMC	128	5	5	62.5	40	30	24
K	Three (C2-3, C3-4, C6-7)	LR-PRP	-	3	3	37.5	42.5	0	6
L	Three (C4-5, C5-6, C6-7)	BMC	71	0	1	0	22.5	70	6
M	One (C5-6)	BMC	40	1	1	30	10	85	3
N	Two (C4-5, C5-6)	LR-PRP	-	3	5	35	52.5	50	12
O	Two (C5-6, C6-7)	BMC	127	4	3	45	30	50	3
P	One (C4-5)	BMC	238	3	3	30	0	95	13
Q	Two (C4-5, C5-6)	LR-PRP	-	6	6	57.5	57.5	30	12
R	One (C5-6)	BMC	237	5	4	57.5	57.5	0	18
Mean			125.5	3.4	2.4	39.1	28.2	60.0	12.2
Standard deviation			73.0	2.2	2.0	19.2	21.4	33.1	7.7

Eight patients were treated with intradiscal injections of LR-PRP and ten with BMC. Although blood preparations were not characterized for this study, the laboratory process used to prepare LR-PRP recovers an average of 80% of platelets, corresponding to an estimated dose of 2.4 to 7.2 billion platelets per mL of LR-PRP injected, assuming a normal blood range (150 to 450 million platelets per mL). This corresponds to a platelet concentration 16 times baseline and a total of 1.2 to 3.6 billion platelets injected per disk in 0.5 mL of injectate volume. Cell counts on LR-PRP samples (n = 10) prepared using the same methodology indicate that the number of leukocytes increases approximately fourfold compared to that in whole blood. Total nucleated cell counts for BMC ranged from 160 to 474 million per mL (244 ± 94 million per mL), corresponding to 40 to 238 million cells per disc (126 ± 73 million per disc).

**Figure 4:**
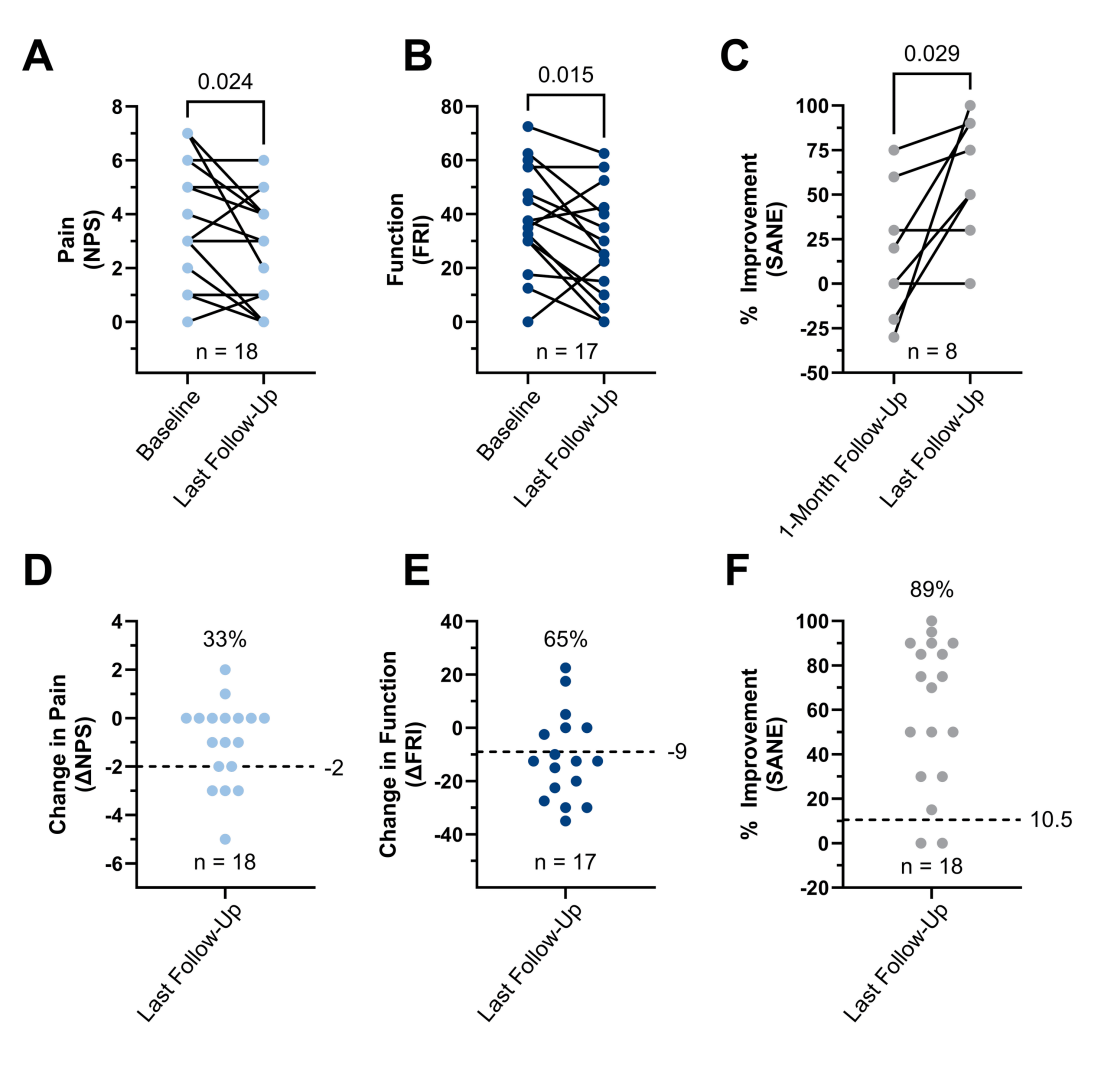
Patient Reported Outcomes Measures (PROMs) Following Intradiscal Injections With Autologous Orthobiologics for Cervical Discogenic Pain. Baseline and last reported follow-up scores for pain (A), function (B), and % improvemnet (C) compared via paired t-test (P<0.05). Change scores for pain (D), function (E), and % improvement (F) alongside the percentage of patients meeting or exceeding previously reported MCIDs (dashed lines).

There were no adverse events reported for any of the patients in this data set. There was no post-procedural bleeding, even in cases where the needle path traversed the jugular vein. The last reported follow-up ranged from three to twenty-four months post-treatment (12.2 ± 7.7 months). A small but significant reduction in NPS scores was observed from baseline to last follow-up (mean difference = -1.0, P = 0.024), with nine of eighteen patients reporting a decrease in pain level, seven reporting no change, and two reporting increased pain (Figure 4A). A majority of patients (14 of 18) reported functional improvement, corresponding to a significant decrease in FRI scores (mean difference = -10.9, P = 0.015) (Figure 4B). While SANE scores significantly improved over the one-month follow-up (mean difference = 43.75, P = 0.029), only eight patients provided 1-month data (Figure 4C). Only 33% of patients met or exceeded the MCID for NPS, whereas 65% and 89% of patients met or exceeded the MCID for FRI and SANE, respectively (Figure 4D-F).

## Discussion

Cervical discogenic pain is a debilitating condition that often requires innovative treatment approaches. This case series explores the safety and potential effectiveness of intradiscal injections, including BMC and high-concentration LR-PRP, as part of a comprehensive cervical FSU approach. We have also described two variants of the cervical intradiscal procedure technique that combine the use of ultrasound and fluoroscopic guidance to increase both accuracy and safety. The injection techniques used were similar to two separate case reports by Lam et al. for preferred and alternative approaches [9,10]. The addition of musculoskeletal ultrasound to the traditional palpation-guided or fluoroscopy-only approaches increases the safety and accuracy of the cervical intradiscal procedure [21]. There were no procedural complications, suggesting that the techniques used have a good safety profile, but conclusions are limited by the small sample size.

There are several studies on the use of autologous PRP and BMC for lumbar discogenic pain, suggesting similar benefits and mechanisms of action in the cervical discs [7,8]. PRP contains various growth factors in its alpha granules, such as transforming growth factor beta and platelet-derived growth factor, that may help collagen synthesis in the annulus and attract healing cells [7]. Another proposed mechanism of action with BMC is the presence of mesenchymal stem cells, also known as medicinal signaling cells (MSCs), which can help to heal annular tears, resulting in less irritation to the sinuvertebral nerves that innervate the outer annulus [22]. They may also decrease disc degeneration and endplate changes [23]. Moreover, injection of MSCs into the lumbar disc has been shown to reduce some protrusions and bulging, leading to less spinal nerve root irritation and reduced radicular symptoms [24,25]. The importance of platelet and cellular dosing has been demonstrated in recent publications in relation to clinical outcomes for the treatment of different musculoskeletal pathologies [7,8,26-30]. Here, we report very high platelet and total nucleated cell counts (TNCC), and thus did not observe variability in outcomes across platelet doses or TNCC. A larger study with more variability in platelet and/or TNCC counts may be helpful to elucidate a potential dose-response to therapy. A limitation of this study was the lack of direct measurement of baseline platelet levels, so the numbers reported are estimations based on an assumed normal range, as all participants were healthy adults.

Despite significant research on PRP and BMC for lumbar spine discs, there is limited research on the use of autologous orthobiologics for cervical intervertebral disc conditions. Only two case reports on cervical intradiscal injection of PRP, with a combined total of two patients, have been published [9,10]. Our research group previously published a similar case series on PRP and prolotherapy utilizing an FSU approach without intradiscal injection that showed significant reductions in pain, improved function, and improved SANE scores up to two years post-treatment [12]. The functional spinal unit approach makes it difficult to create a well-controlled randomized trial, but it represents real-world patients who often have complex and variable pathologies requiring a more personalized treatment plan. The results of this case series suggest that intradiscal injections within a cervical FSU approach may offer a safe and beneficial treatment option for many patients with cervical discogenic pain and other cervical spine injuries that have failed standard-of-care therapy. Most (14 of 18) patients reported greater than 50% overall improvement, and 65% met the MCID for function. Reduction in pain was not as dramatic, likely due to the relatively small sample size, limited follow-up period, and the fact that most of the study participants reported lower baseline pain levels, making further reduction harder to demonstrate. However, the study’s limitations, including its small size, lack of a control group, variable pathologies, multiple injection targets, and retrospective nature, necessitate larger prospective controlled trials for further evaluation.

## Conclusions

Intradiscal injection(s) of high-concentration LR-PRP or BMC, combined with an FSU treatment approach, show promise in safely improving function and satisfaction in patients with cervical discogenic pain and other cervical musculoskeletal pathologies. Further investigation is warranted.
